# Effect of vitamin E supplementation on orthodontic tooth movement in Wistar rats: a prelimary study

**DOI:** 10.12688/f1000research.25709.3

**Published:** 2020-12-07

**Authors:** Erliera Sufarnap, Darmayanti Siregar, Yumi Lindawati

**Affiliations:** 1 Department of Orthodontics, Universitas Sumatera Utara, Medan, North Sumatera, 20155, Indonesia; 2 Department of Dental Public Health, Universitas Sumatera Utara, Medan, North Sumatera, 20155, Indonesia; 3 Department of Oral Biology, Universitas Sumatera Utara, Medan, North Sumatera, 20155, Indonesia

**Keywords:** Orthodontic tooth movement, vitamin E, tooth movement distance, osteoblast, osteoclast.

## Abstract

**Background**: Tooth movement induced by the application of orthodontic force was initiated by inflammatory process. Studies have shown that vitamin E has an anti-inflammatory and antioxidant properties which perhaps could inhibit the tooth to move. This study aimed to evaluate the effect of vitamin E supplementation on orthodontic tooth movement in Wistar rats.

**Methods**: Wistar rats (n=56) were divided into two groups. Group 1 served as the control groups, while group 2 was given vitamin E for 14 days before application of orthodontic force. Each group was divided into four subgroups (n=7), corresponding to the number of days orthodontic force lasted, i.e. 0, 1, 3, 7 days. At each of these four time points, distance measurements and quantity of osteoblasts-osteoclasts were measured in each rat.

**Results:** Tooth movement distance was increased for group 2 than group 1 for all time intervals, but this difference was only statistically different on day 3 ( *p*=0.001). For both groups, tooth movement was significantly different between each time interval in each group ( *p*=0.041). The mean number of osteoblast cells was increased for group 2 compared to group 1 for all time intervals (p<0.05), but was not significant different between time intervals ( *p*=0.897). The number of osteoclasts was not significantly different between groups, but it was statistically different between time intervals (p=0.004).

**Conclusion:** The outcome of this study demonstrated that group 2  resulted a better tooth movement compared to group 1 and significantly found on day 3, based on the distance measurement. The osteoclast cell numbers were the same within both control groups, whilst  the number of osteoblast cells in group 2 was significantly higher than those in group 1.

## Introduction

Tooth movement is induced by the application of orthodontic force characterized by bone and periodontal tissue remodelling. Orthodontic force also alters periodontal tissue vascularity and blood flow, resulting in the local synthesis and release of various molecules such as neurotransmitters, cytokines, growth factors, colony-stimulating factors and arachidonic acid metabolites ^1^.

Bone remodelling is a process that enables tooth movement. It involves bone-reabsorption by osteoclasts on the pressure site and bone-formation by osteoblasts on the tension site ^2, 3^. Osteoclasts are multinucleated cells, irregular in shape with a process originating from Howship’s lacunae ^4^. They stimulate bone resorption by creating cavities in the bone known as lacunae that will be filled by osteoblast cells ^3^. According to Mavragani *et al.*, the cellular process of osteoclast proliferation has been used as important indicators in evaluating the level of tooth movement ^5^. Osteoblasts are mononuclear cells that originate from mesenchymal stem cells in bone marrow. Mature osteoblasts form the osteoid by synthesizing collagen and non-collagen proteins ^6^.

According to Burstone in Asiry’s citation, there are three phases of orthodontic tooth movement, which consists the initial, lag and postlag phases ^7^. The initial stage of orthodontic tooth movement stimulates an inflammatory response involving cells and blood vessels in periodontal ligaments as well as chemical mediators. In response to mechanical stress caused by the application of orthodontic force, substances such as cytokines and enzymes are released ^2, 8^. Interleukin-1β is a pro-inflammatory cytokine that facilitates fusion and activation of osteoclasts, and encourages early bone resorption ^9^.

Studies have shown that vitamin E has anti-inflammatory properties, which helps suppress damaging effects of oxygen free radicals in cells during bone formation ^10^. Previous studies carried out by Esenlik *et al.* and Xu *et al.* suggest that vitamin E supplementation may alter cytokine production; vitamin E supplement maintains normal bone remodelling in young animals and increases bone mass by decreasing the concentration of free radicals which suppress bone formation ^11, 12^.

Since orthodontic tooth movement is mediated by inflammation process (bone remodelling of cells and chemical mediators, cytokines and growth factors), it is possible to hypothesize that vitamin E will reduce the tooth movement with its anti-inflammatory effects but it also has a positive effect on bone formation process. The purpose of this study was to evaluate the effect of vitamin E supplementation on tooth movement distance and osteoblast and osteoclast cells in Wistar rats. Mice and rats are mammals that have a reasonably comparable metabolism to humans, which can be used for biological-cellular mechanism analysis in orthodontic tooth movement ^13^.

## Methods

### Animals

This article was reported in line with the ARRIVE guidelines. The study was an *in-vivo* quasi experiment, which was approved by the Animal Research Ethics Committee, Department of Biology - Faculty of Mathematics and Science, Universitas Sumatera Utara (No. 0128/KEPH-FMIPA/2019).

A total of 56 healthy male, four to five-months old, Wistar rats, weighing 150–250 grams, were used in this study. The Wistar Rats came from the same breeding farm (Deli Serdang, North Sumatera, Indonesia) in two cycles.

The rats were adapted to their environment for 7 days before the experiment start. They were nurtured at the Animal House at Faculty of Mathematics and Science, Universitas Sumatera Utara in polycarbonate cage, which measured 480 mm × 265 mm × 210 mm. Each cage had wood shavings on the floor, and contained 3 or 4 animals, which were marked for each subgroup. Rats were chosen for each group by simple random sampling.

Low light to dark cycle was maintained for a minimum 12 hours at 25–30°C for room temperature within the experiment period. The rats were given a standard pellet diet. All conditions served to produce the optimum condition of the rats’ habitat ^14^. A rubber separator was inserted between maxillae’s incisors to produce non-invasive experiments. Anaesthetic was used to euthanize the rats at the end of the experimental procedure.

### Experiment

Wistar rats (n=56) were divided into two groups. Each group was then divided into four subgroups (n=7), corresponding to the number of the days orthodontic force lasted, i.e. 0, 1, 3, 7 days. Vitamin E or tochoperols (VE) were reportedly able to modulate the estrogen receptor-β (ERβ) therefore male rats were choosen as subjects for this study ^15^. The sample size of each subgroup was decided by Sastroasmoro and Ismael’s formula for hypothetical analysis between independent variables ^16^.

Subgroups were chosen based on the rats’ social behaviours. Hyperactive rats were chosen to be in the same cage, separately to rats with a more passive behaviour. These conditions avoided any anxiety social-related behaviour between rats in the cage within the experiment. For each experiment, a researcher who was blind to the experiment chose a sample randomly from each cage.

Group 1 were the control group and were given water orally as a placebo. The rats’ tail was marked with black pen. Group 2 were the experimental group and were given VE (dl-α-Tocopheryl Acetate; Sanbe, Indonesia) at a dosage of 60 mg/kg, orally using gavage needle. The dose chosen was based on a research that had been done by Norazlina *et al*. ^17^. The group 2 rats’ tail marked with red pen.

**Figure 1.**
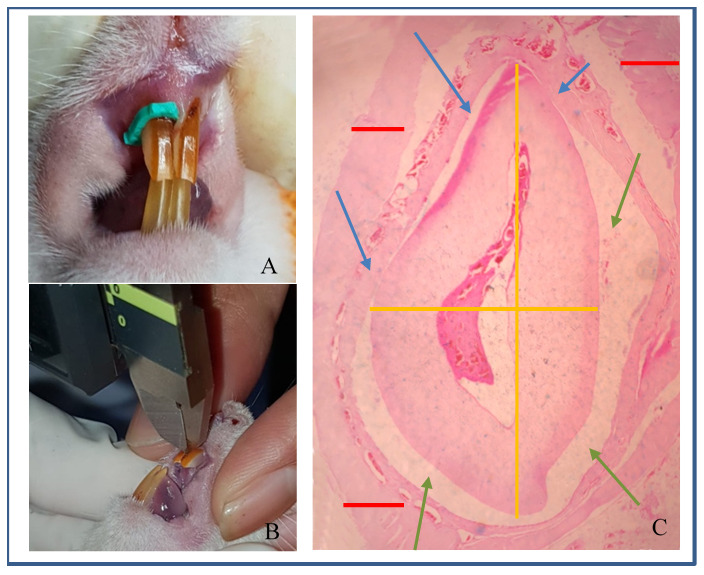
( **A**) Rat separator; ( **B**) Distance measurement; ( **C**) Microscopic of whole teeth at 40x magnification. **Lines**, Yellow=teeth; Green=periodontal ligament; Red=alveolar bone. **Arrows,** Blue=pressure side; Green=tension side.

Water and VE were given every day at 8am, for 14 days before and continued after application of orthodontic force. After 14 days, orthodontic force was applied to each rat in both groups by addition of a rubber separator to one of the maxilla incisors ( Figure 1A). This administration of orthodontic force applied were carried out before daily water and VE feeding. This procedure counted as the baseline time of the experiment. At each of these four time points distance measurements and quantity of osteoblasts-osteoclasts were measured (see section below).

At end of each experiment period, the dosage of ketamine® at 80mg/kg of body weight and xyla® (Interchemie, Holland) at 10mg/kg of body weight was used to euthanised each rat by cardiac puncture methods for further research with blood analysis.

### Outcomes

Tooth movement was measured using a digital calliper (Mitutoyo, Japan) was used to measure the distance between maxilla incisors at mesial cervical (Moorrees method) immediately after removal of the rubber separator ( Figure 1B) ^18^.

The pre-maxillae were dissected and fixated in 10% formalin for 24h, and decalcified with rapid-decalsifier, Nitric acid 10% (Aurona Scientific, Singapore) for 10–14 days. The embedded blocks were trimmed using a Leica microtome (Leica, Germany) into 5µm sections. Histological sections were stained with haematoxylin-eosin and were examined using Olympus CX21 light microscope at 400x magnification to analyse the number of cells within five fields of view for each measurement.

A pressured site on the distal of the tooth exhibited the region of interest (ROI) for a quantitative measured for osteoclast analysis which were described as a narrow area between teeth and alveolar bone where the tooth tended to move, and this site was used for osteoclast analysis. A tension site on the mesial of the tooth exhibited the ROI for a quantitative measured for the osteoblast analysis which were described as a wide area between teeth and alveolar bone where the tooth was left out, and this site was used for osteoblast analysis ( Figure 1C).

The histological methods to identify the osteoclasts-osteoblasts include visualization of the cell characteristics and location. Osteoblast describes as a basophilic cuboidal or polygonal mononuclear cells which located on bone surfaces ^19^. Osteoclast describes as a giant, multinucleated cell which has an average of 3–20 nucleus, the shape tends to be oval. Osteoclasts are very motile at various sites along the bone surface, it describes the varied appearance of these cells ^20^.

### Statistical analysis

IBM-SPSS (Statistical Package for Social Sciences), version 26.0, was used for statistical analysis. Independent t-test and Mann-Whitney test were used to analyse the difference between the two main groups and the inter-rater relialibility (IRR) determination were analysed with Cronbach’s alpha level. General Linear Model-Repeated Measures (ANOVA GLM-RM) and Friedman analysis were used to analyse the difference between time intervals. Significant differences were determined at p<0.05.

## Results

Throughout the feeding of VE supplementation, all rats were habituated to reduce their stress-related disturbances and they seemed to be in a good condition during administration of VE, and no rat had undergone for toxicity and neither had been death within the experimental period.

Determination of osteoblast’s and osteoclast’s quantity were done by 2 examiners, a senior anatomical pathologists and an orthodontist. Cronbach’s alpha interpretation to inter rater relialibility (IRR) were 4 samples from group 1 for each time’s period, osteoblast’s level for alpha was -0,181 (ANOVA, *p-value*=0,521) and osteoclast’s level for alpha was 0,7 (Friedman, *p-value*=0,001). Mean value from both rater were decided to be the amount numbers for this data analysis.

Tooth movement distances were greater in group 2 compared to group 1 at each time point ( Table 1). This difference was only statistically significant on day 3 (p=0.001). For both groups, tooth movement was significantly different between each time interval in each group ( *p*=0.041). After day 3, movement for group 1 reduced, while for group 2, this continued to increase until day 7.

**Table 1.** Comparison of tooth movement distance between Group 1 (control) and group 2 (vitamin E treatment (n=7/subgroup (day)). Data are presented as mean±SD.

Day	Tooth movement (mm)		*P* value ^a^	*P* value ^b^
	Group 1	Group 2		
0	0.00±0.00	0.00±000	Baseline	0.041
1	0.25±0.05	0.31±0.13	0.486	
3	0.22±0.12	0.50±0.11	0.001	
7	0.37±0.20	0.55±0.22	0.1373	

p<0.05 – statistically significant. ^a^Independent t-test; ^b^ANOVA GLM-RM

**Figure 2.**
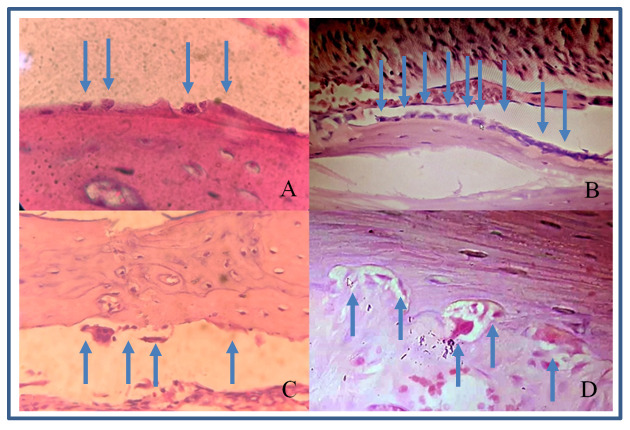
Osteoblasts and osteoclasts in rat alveolar bone at 400x magnification. ( **A**) osteoblasts in group 1 (control); ( **B**) osteoblasts in group 2 (vitamin E treatment); ( **C**) osteoclasts in group 1; ( **D**) osteoclasts in a Howship’s lacuna in group 2.

The number of osteoblasts in group 2 were higher compared with group 1 at each time point ( Figure 2A and B; Table 2). These differences were statistically significant (p<0.05). Group 2 showed increased osteoblasts starting from day 0 to day 3, while group 1 had decreased osteoblast after day 3.

**Table 2.** Comparison of number of osteoblasts between Group 1 (control) and group 2 (VE) (n=7/subgroup (day)). Data are presented as mean±SD.

Day	Number of osteoblasts (n)		*P* value ^a^	*P* value ^b^
	Group 1	Group 2		
0	5.14±1.34	9.21±3.21	0.012	0.001
1	5.29±1.71	9.36±2.38	0.003	
3	3.86±1.94	10.14±3.53	0.004	
7	5.04±0.95	8.43±1.02	0.002	

p<0.05 – statistically significant. ^a ^Mann-Whitney test; ^b ^Friedman analysis

The number of osteoclasts in group 2 were higher than group 1 except on day 1, but the differences were not significant statistically ( Figure 2C and D; Table 3).

**Table 3.** Comparison of number of osteoclasts between Group 1 (control) and group 2 (VE) (n=7/subgroup (day)). Data are presented as mean±SD.

Day	Number of osteoclasts (n)		*P* value ^a^	*P* value ^b^
	Group 1	Group 2		
0	0.89±0.48	1.18±0.47	0.393	0.016
1	1.86±0.93	1.79±0.77	0.797	
3	1.07±0.91	1.68±0.67	0.109	
7	1.82±1.01	2.18±0.93	0.172	

p<0.05 – statistically significant. ^a^Mann-Whitney test; ^b^Friedman analysis

## Discussion

Orthodontic force causes gradual compression on the periodontal ligament, which leads to circulatory disorders, such as ischemia and hypoxia in the early stage of orthodontic tooth movement ^21^. Hypoxia and compression caused by orthodontic force stimulate the production of reactive oxygen species and free radicals, which contribute to cellular and tissue damage, especially damaging lipid peroxidation chains ^22^. Vitamin E is a strong biological antioxidant that has several functions: scavenges free radicals, which inhibit lipid peroxidation and inflammation; protects ischemic tissue and hypoxia; provides immunostimulation ^11, 23^. Norazlina *et al.* observed the effect of vitamin E supplementation on bone metabolism in mice treated with nicotine. Their study results suggested that vitamin E can increase trabecular bone formation and prevent bone calcium loss by reducing pro-inflammatory cytokines ^17^. McGavin *et al.* concluded that vitamin E will have an effect to the plasma alpha tocopherol levels minimum at weeks 4 untill 6 from the dietary intake of vitamin E resources whilst from the supplement of 220 IU/day, the levels significantly increased within 2 weeks and remained untill weeks 8 ^24^.

In the present study, it can be seen that both groups showed increased tooth movement distance as well as increase in the number of osteoclast and osteoblast cells on day 1. This is due to the initial phase of tooth movement after application of orthodontic force ^25^. This phase occurs 24 hours to 48 hours after application of orthodontic force on teeth ^3^.

Our results showed that the number of osteoclasts is higher in group 2 compared to group 1 although the difference was not statistically significant. Miresmaeili *et al.*, in their study on the effect of vitamin C to orthodontic tooth movement, found that osteoclast numbers were significantly higher in the vitamin C group, which hence accelerates tooth movement ^26^. Kale *et al.*, in their research on vitamin D injection, observed a significant amount of Howhip’s lacunae in resorption cavity as a result of osteoclast’s activity ^27^. Future research is required to observe the comparison between Howship’s lacunae and osteoclasts numbers.

In our study, there were statistically significant differences in the mean number of osteoblast cells between both groups at each time observed. The osteoblast cell numbers significantly higher from the baseline period since the rats already intervened with vitamin E 14 days prior the experiment time. McGavin mentioned that vitamin E supplementation would increased the plasma alpha tocopherol levels minimum of 2 weeks ^24^. Kawakami and Takano-Yamamoto demonstrated an increased osteoclast and osteoblast number with local injection of 1,25-dihydroxyvitamin D3 in the submucosal palatal area of rats subjected to tooth movement on day 7. Increased osteoblast counts were observed on day 14 ^23, 28^. In another study, Feresin *et al.* reported that the formation rate and bone volume increased significantly by 65% in rat bone, who were given a vitamin E diet compared to the control group. Their result indicated that a vitamin E diet was able to increase the process of mineralization and bone formation mediated by osteoblast cells ^29^. Diravidamani *et al.* stated that many drugs that are used to reduce pain had effects on orthodontic tooth movement. Further research should be done to observe vitamin E on pain regulation, because it has anti-inflammatory effect, which is assumed to reduce pain in orthodontic treatment ^10, 30– 33^.

The force mechanism from the separator used in our study was static and the elasticity from the separator is easily lost due to saliva acidity (pH), food and chewing process; a the force of a rubber separator will be reduced by 50–55% within 24 hours ^33^. This is a limitation of our study, as we wanted to analyse for a longer time and with a larger force. The aim of our study was to see the orthodontic tooth movement and not stabilization, so we decided to observe the orthodontic movement within the initial phase, and not all phases until the lag phase.

The Cronbach’s alpha test for the inter-rater relialibility (IRR) analysis showed a weak result correlation of osteoblast’s level which had a reverse and a “poor” correlation between both agreement. Whilst the the osteoclast’s level had an acceptable agreement between IRR. This is also the limitation of this study, further analysis supposed to be complete with specific biomarker for osteoblast and also for osteoclast immunohistochemistry staining which is an important tool to determine the bone resorption and bone remodelling for scientific research which are not determinable by haematoxylin-eosin staining alone ^34^. These limitations of the study made this study was designed as a preliminary study.

## Conclusions

Our findings demonstrated that which could be seen in the distance measurement. The osteoclast cell numbers have the same within control groups, whilst the number of osteoblast cells in the VE supplementation group was significantly higher than those in control group. Our hypothesis on the effect of VE supplementation on tooth movement were accept the null hypothesis in osteoclast count which meant that it didn’t show inhibitted effect in the tooth to move.

## Data availability

### Underlying data

Open Science Framework: Methods, Figures, and Results from "Effect of Vitamin E Supplementation on Orthodontic Tooth Movement in Wistar Rats, https://doi.org/10.17605/OSF.IO/3S4QB ^35^.

### Reporting guidelines

Open Science Framework: ARRIVE checklist for ‘Effect of vitamin E supplementation on orthodontic tooth movement in Wistar rats’, https://doi.org/10.17605/OSF.IO/3S4QB ^35^.

Data are available under the terms of the Creative Commons Attribution 4.0 International license (CC-BY 4.0).
