# Supplementary material for: Effect of vitamin E supplementation on orthodontic tooth movement in Wistar rats: a prelimary study
Source: F1000Res. 2020 Dec 7;9:1093. Originally published 2020 Sep 4. [Version 3] doi: 10.12688/f1000research.25709.3 (PMC7687191; doi:10.12688/f1000research.25709.3)
Supplement: Peer review history

# Peer review: Reviewer response for version 2

**DOI:** 10.5256/f1000research.30027.r73171

**Published:** 2020-11-24

**Authors:** Hiroyuki Kanzaki

**Affiliations:** 1 Maxillo-oral Disorders, Tohoku University Hospital, Sendai, Japan; 2 Department of Orthodontics, School of Dental Medicine, Tsurumi University, Yokohama, Japan

Thank you for an excellent revision of the manuscript. The reviewer felt that this revised manuscript is appropriate for next step, except for one issue. It is no scale bar at figure 2.

 If your lab is performing in vitro assay and has hemotocytometer, you can calibrate the microscope scale by using the hemotocytometer. If your lab has no hemotocytometer, microscope calibration slide would help the calibration. Microscope calibration slide is around $10.00, and not so expensive. I recommend you to set the scale in figure 2 by using microscope calibration slide...

Is the work clearly and accurately presented and does it cite the current literature?

No

If applicable, is the statistical analysis and its interpretation appropriate?

Partly

Are all the source data underlying the results available to ensure full reproducibility?

Partly

Is the study design appropriate and is the work technically sound?

No

Are the conclusions drawn adequately supported by the results?

Partly

Are sufficient details of methods and analysis provided to allow replication by others?

No

Reviewer Expertise:

bone biology

I confirm that I have read this submission and believe that I have an appropriate level of expertise to confirm that it is of an acceptable scientific standard.

# Response

**Published:** 2020-11-28

**Authors:** Erliera Sufarnap

**Affiliations:** Universitas Sumatera Utara, Indonesia

**Dear Prof. Hiroyuki Kanzaki;**

 Maxillo-oral Disorders, Tohoku University Hospital, Sendai, Japan;  Department of Orthodontics, School of Dental Medicine, Tsurumi University, Yokohama, Japan  

 We send a thousand thanks, gratitudes and appreciations of your knowledge and advices.

 We currently order the microscope calibration scale and the eyepiece graticule for the Olympus microscope. Unfortunately within this pandemic, the university’s laboratorium were closed and some of them switched to ‘other’ function for the pandemic purposes. We were terribly ask for the forgiveness. Soon after we receive the calibration sets or the condition of the lab’s will clear and clean, we try our best to complete it regardless to the unknown of time prediction.

 Once again, I apologize for this matter and thanking you so much for the valuable knowledge for our research.

# Peer review: Reviewer response for version 1

**DOI:** 10.5256/f1000research.28373.r71955

**Published:** 2020-09-25

**Authors:** Hiroyuki Kanzaki

**Affiliations:** 1 Maxillo-oral Disorders, Tohoku University Hospital, Sendai, Japan; 2 Department of Orthodontics, School of Dental Medicine, Tsurumi University, Yokohama, Japan

In this manuscript, the authors examined the effect of vitamin E (VE) supplementation on orthodontic tooth movement (OTM). They found that VE augmented OTM only at day-3. The number of osteoblasts was increased by VE at any time point, though the number of osteoclasts was stable irrespective of VE. This manuscript seems at the development stage, and there are some concerns and they are discussed below.

**Major:**

1. Increased osteoblast at day 0 in group-2.
                Why was there a statistically significant difference on day-0?
2. Model of OTM
                The reviewer felt that the model the authors used is not suitable for the model of OTM, due to excessive heavy force by elastic separation band. Other models such as the use of super-elastic coil spring between molar and incisor would be suitable.

 

**Minor:**

1. Please add the scale in figure 2.
2. Vitamin E dosage
                Please describe how the authors decided the dose of vitamin E in this experiment.

Is the work clearly and accurately presented and does it cite the current literature?

No

If applicable, is the statistical analysis and its interpretation appropriate?

Partly

Are all the source data underlying the results available to ensure full reproducibility?

Partly

Is the study design appropriate and is the work technically sound?

No

Are the conclusions drawn adequately supported by the results?

Partly

Are sufficient details of methods and analysis provided to allow replication by others?

No

Reviewer Expertise:

bone biology

I confirm that I have read this submission and believe that I have an appropriate level of expertise to state that I do not consider it to be of an acceptable scientific standard, for reasons outlined above.

# Response

**Published:** 2020-10-01

**Authors:** Erliera Sufarnap

**Affiliations:** Universitas Sumatera Utara, Indonesia

Dear, Assoc. Prof. Hiroyuki Kanzaki, Maxillo-oral Disorders, Yohoku University Hospital, Sendai, Japan

 Thank you for your kind attention and assistance in reviewing our manuscript with the precious advices for the manuscript and our further research. We have revised the manuscript according to some inquiries you have been suggested. The following are our comprehensive comments to the review after revised.

**Major :**

 1. “In this manuscript, the authors examined the effect of VE supplementation on OTM. They found that VE augmented OTM only at day 3”. 

**Comment : **The distance measurement showed that the distance of the group 2 (VE) seemed wider for all time point of observation, but unfortunately we found significantly difference only on day 3 since the initial phase was happened within this period of time. 

 2. “The number of osteoblasts was increased by VE at any time point since the baseline time (day-0)”

**Comment : **VE already intervened 14 days prior the orthodontic separator insertion based on McGavin et al. study ^24^. The authors wanted to observe the OTM after the VE circulated to the rats body since VE is one of the regular vitamin to be intake daily.

 3. “This manuscript seems at the development stage and there are some concerns suppose to be discussed below” 

**Comment : **Yes Indeed, we discuss this is as our limitation of the study on the discussion chapter, moreover we added the Title of this manuscript become “A preliminary study”.

 4. Model of OTM: “The reviewer felt that the model the authors used is not suitable for the model of OTM, due to excessive heavy force by elastic separation band. Other models such as the use of super-elastic coil spring between molar and incisor would be suitable”.

**Comment :** Yes Indeed. For further research we should effort ourself to have more controllable and continuously force of the mechanic. We thank you again for this valuable insight and definitely we will be consider in our future works.

    

**Minor:**

 1. Please add the scale in figure 2

**Comment : **The Olympus CX21 light microscope we used did not have any scale bar and so that the slide we used. We were sorry for our laboratory limitation. I have tried so hard to put in to ImageJ and converted it, but still it could not be processed properly. So, still I couldn’t give the answer your inquiry. 

 2. VE dosage 

**Comment** : We have edited and added to the experiment chapter

 We thank you again for your kindness for the established and valuable insight of the reviews.

# Peer review: Reviewer response for version 1

**DOI:** 10.5256/f1000research.28373.r70989

**Published:** 2020-09-09

**Authors:** Ananto Ali Alhasyimi

**Affiliations:** 1 Department of Orthodontics, Faculty of Dentistry, Gadjah Mada University (UGM), Yogyakarta, Indonesia

It’s been an honor for us to review the manuscripts. First of all, please allow me to congratulate the authors for endeavoring to undertake this study which I found very interesting and valuable as a recommendation for further study in the orthodontics field especially in the acceleration of orthodontic tooth movement area. We have been reviewing throughout the manuscript. The following are our comments related to the manuscript.

**General Comment:**

- The title and topic are quite interesting for journal scope and discover a novelty especially for developing natural materials to accelerate orthodontic tooth movement.
- Paper is well-organized and easy to follow.
- To improve the readability, It is recommended that the text is given an English language edit as many of the sentences might be misunderstood and some of typos were found. I suggest a revision of the English grammar structures by an expert editor in revising manuscripts.

**Abstract**:

- The author mentioned: “Tooth movement induced by the application of orthodontic force is facilitated by bone remodeling cells and chemical mediators. Vitamin E has anti-inflammatory properties, which helps in suppressing the damaging effects of oxygen free radicals in cells during bone formation”. As we know that inflammation is a part of the orthodontic tooth movement, so it is better to find another logic to connect the reason for using Vitamin E in this study.
- It stated: “Conclusion: Present outcomes demonstrate that vitamin E contributes to faster tooth movement compared to control group. It also stimulates more bone formation without reducing the bone resorption”. The author only evaluates the osteoclast and osteoblast number and did not analyze bone formation nor bone resorption indicator. It is better to write a conclusion based on what the author did.

**Introduction**:

- At the end of the introduction part please add some of the hypotheses.

**Methods:**

1. Author used healthy male Wistar ratsà what is the rationale? Please explain shortly in the manuscript.
2. A dosage of 60 mg/kg Vitamin E was used in this study, what is the rationale? Please explain shortly in the manuscript.
3. Authors have chosen HE staining for labeling osteoclast and osteoblast number but did not use any specific marker for osteoblasts (e.g. Von-kossa or Alkaline phosphatase.) and osteoclast (TRAP), rather relied on visual analyses and counting of cells. This method of counting cells is somewhat arbitrary. There are a number of markers available for osteoblasts – osteoclasts using these biomarkers will strengthen this study.
4. Author should add the special characteristic of osteoclast-osteoblast (e.g. osteoblast was demonstrated as a cuboidal shape cell, characterized by a single, large, deep blue-purple nuclei, and found on the edge surfaces of alveolar bone) to minimize the bias and add the region of interest (ROI) since ROI is the key element in both number counts and distribution. Therefore, it is of utmost importance to show where were these ROIs.
5. A blinded evaluator was responsible for determination of the score and had undergone a training exercise involving the calculation of the Kappa coefficient for the determination of intra-examiner agreement, what about the result? Please write it whether it is satisfactory or not.

 

**Results **

- General results were not reported. What happens with the general health of the animal during the administration of Vitamin E (e.g.general toxicity, edema, deaths affect the body weight of animals?)

**Discussion**

- Fairly well written with minor typographical errors.

**Conclusion **

- The author only evaluates the osteoclast and osteoblast number and did not analyze bone formation nor bone resorption indicator. It is better to write a conclusion based on what the author did.

Is the work clearly and accurately presented and does it cite the current literature?

Yes

If applicable, is the statistical analysis and its interpretation appropriate?

Yes

Are all the source data underlying the results available to ensure full reproducibility?

Yes

Is the study design appropriate and is the work technically sound?

Yes

Are the conclusions drawn adequately supported by the results?

Partly

Are sufficient details of methods and analysis provided to allow replication by others?

Yes

Reviewer Expertise:

orthodontic biomaterials

I confirm that I have read this submission and believe that I have an appropriate level of expertise to confirm that it is of an acceptable scientific standard.

# Response

**Published:** 2020-10-01

**Authors:** Erliera Sufarnap

**Affiliations:** Universitas Sumatera Utara, Indonesia

Dear Dr Ananto Ali Alhasyimi, Universitas Gadjah Mada, Sleman, Indonesia

Thank you for your kind assistance in reviewing our manuscript becoming very marvelous manuscript with your advices at many chapters. We have revised the manuscript according to some inquiries you have been suggested. Finally we add the title become ‘A preliminary study’ which suppose to be our limitation of this research and would be continue to further research which we mentioned on discussion part. Several additional references already provided to consolidated theories which were needed. The following are our comprehensive comments to the review after revised.

1. Abstract
  1. “As we know that inflammation is a part of the orthodontic tooth movement, so it is better to find another logic to connect the reason for using Vitamin E in this study”.
    -  : We have edited the reason of using VE in this study
2. The author only evaluates the osteoclast and osteoblast number and did not analyze bone formation nor bone resorption indicator. It is better to write a conclusion based on what the author did.
  -  : We have edited the conclusion based on the variables we observed without any other conclusion.

2. Introduction : “  At the end of the introduction part please add some of the hypotheses”.

**Comment** : We have edited our hypothesize more pointedly.

3. Methods :  

a. “Author used healthy male Wistar rats what is the rationale? Please explain    shortly in the manuscript.”

**Comment** : We were sorry, we’ve missed the detail to explain why. We edited and gave a reason that VE able to modulate the estrogen (hormone) and a reference had been added to strengthen this reason.

b. “A dosage of 60 mg/kg Vitamin E was used in this study, what is the rationale? Please 

explain shortly in the manuscript.

**Comment** : The dose chosen was based on a research that had been done by Nur Azlina et al. ^17^_._

c. “There are a number of markers available for osteoblasts – osteoclasts using these biomarkers will strengthen this study”. 

**Comment** : We mentioned changes in the title and discussion chapter as our limitations of the study

d.  Author should add the special characteristic of osteoclast-osteoblast to minimize the bias and add the region of interest (ROI) since ROI is the key element in both number counts and distribution. Therefore, it is of utmost importance to show where were these ROIs.

**Comment** :  Thank you Doctor, We completed and added additional references to mention about the characteristic of osteoblasit and osteoclast and expalained about the specific ROI.

e.  A blinded evaluator was responsible for determination of the score and had undergone a training exercise involving the calculation of the Kappa coefficient for the determination of intra-examiner agreement, what about the result? Please write it whether it is satisfactory or not.

**Comment** : To be honest, the level for IRR results have not very satisfactory result. The observer decided to use the “main value” from both raters as the datas to be analysed. And we also editted and added this results as our limitation of the study at the discussion chapter.

4. Results : General results were not reported. What happens with the general health of the animal during the administration of Vitamin E (e.g.general toxicity, edema, deaths affect the body weight of animals?)

**Comment** : We have edited this inquiry to the result’s chapter.

5. Discussion : “Fairly well written with minor typographical errors”.

**Comment**  : Thank you for the review and we already rechecked and changed.

6. Conclusion :  “The author only evaluates the osteoclast and osteoblast number and did not analyze bone formation nor bone resorption indicator. It is better to write a conclusion based on what the author did”.

**Comment** : We have edited the conclusion based on the variables we observed without any 

     other conclusion.

Thank you for the established reviews and the valuable insight for our manuscript.
